# Editorial: Transcranial Magnetic Stimulation (TMS) in motor control and motor rehabilitation: current trends and future directions

**DOI:** 10.3389/fnhum.2025.1595762

**Published:** 2025-04-07

**Authors:** Elisa Kallioniemi, Yuming Lei, Hang Jin Jo

**Affiliations:** ^1^Department of Biomedical Engineering, New Jersey Institute of Technology, Newark, NJ, United States; ^2^Department of Health and Kinesiology, Texas A&M University, College Station, TX, United States; ^3^Department of Rehabilitation Science, University at Buffalo, Buffalo, NY, United States

**Keywords:** Transcranial Magnetic Stimulation (TMS), motor control, motor rehabilitation, neuromodulation, repetitive TMS

Transcranial Magnetic Stimulation (TMS) has emerged as a promising tool in motor control and rehabilitation. A single TMS pulse applied over the primary motor cortex can depolarize cortical neurons and evoke measurable responses in the muscles (Siebner et al., 2022). When paired stimuli are delivered to the same or different brain areas with varying intervals and intensities, they can assess inhibitory and facilitatory interactions in the cortex, providing insights into the mechanisms underlying motor behavior (Spampinato et al., 2023). Additionally, TMS can be applied as trains of repetitive stimuli at different frequencies, known as repetitive TMS (rTMS). These rTMS protocols modulate cortical excitability at the stimulated site and can also affect remote areas, with effects extending beyond the stimulation period (Fitzgerald et al., 2006; Goldsworthy et al., 2021). In this editorial, we summarize key findings from eight contributing articles that explore the use of TMS as a measurement tool for assessing corticospinal output or a neuromodulatory tool for motor rehabilitation following various neurological conditions. By synthesizing the latest research in this area, we aim to advance our understanding of how TMS can be leveraged to enhance motor outcomes and guide future research directions.

Stroke is one of the most extensively studied populations for testing the effects of rTMS. Due to high variability in chronicity and motor impairments, research has focused on various subgroups of stroke population to study the efficacy of rTMS. For example, Ma et al. studied patients with acute stroke and demonstrated that high-frequency rTMS combined with conventional rehabilitation is more effective in improving clinical functional measures such as walking speed and balance, compared to conventional rehabilitation alone. Fan et al. conducted a comprehensive review and meta-analysis of studies on rTMS in stroke patients at various stages, aiming to identify optimal parameters for enhancing lower extremity motor function. The study found that rTMS effectively improved lower limb motor function across all stages of stroke, but it was particularly more effective in patients in the subacute stage compared to those in the acute or chronic stages. Furthermore, high-frequency rTMS was more effective than low-frequency in improving walking speed, while low-frequency rTMS proved to be the best option for enhancing balance function. The most significant therapeutic effects were typically observed after 20–40 sessions. Qian et al. further investigated the effect of low-frequency rTMS in subacute stroke patients based on their motor evoked potential (MEP) status. The study showed that patients who showed MEPs in an intrinsic hand muscle showed significantly higher improvement in motor function compared to those who did not show MEPs.

rTMS protocols can be combined with other techniques to enhance its efficacy, and such studies have also been conducted in stroke population. Luo et al. reviewed randomized controlled trials to assess the combined effect of rTMS and repetitive peripheral magnetic stimulation (rPMS) on upper limb motor function in stroke patients. The overall findings suggest that high-frequency or low-frequency rTMS combined with rPMS is more effective than rTMS alone or conventional therapy in improving upper limb motor function after stroke. However, the interstimulus interval between rTMS and rPMS were not clearly defined in most of the reviewed studies, highlighting the need for further investigation on this. Another recent approach combined rTMS with electroencephalography (EEG) to enable brain-state dependent delivering of rTMS and deliver it during periods of increased cortical excitability to increase the plasticity (Hussain et al., 2021). Mahmoud et al. applied this concept to investigate the therapeutic potential of EEG-derived rTMS in the rehabilitation of upper limb motor impairment after stroke. While the results did not show significant differences in motor functional outcomes, the study demonstrated the feasibility of this approach in stroke patients.

Stroke and other neurological disorders frequently lead to neurogenic dysphagia, or difficulty swallowing. Li et al. reviewed the role of rTMS as a treatment for dysphagia, alongside other physical therapies. Most studies applied high-frequency rTMS to the cerebellum or pharyngeal motor cortex, typically 5 days per week for 2 weeks. However, stimulation parameters such as exact pulse frequency and pulse count per session varied substantially across studies, as did the methods for assessing swallowing function. Despite these inconsistencies, the review found that rTMS generally improved swallowing function, supporting its potential as a therapeutic approach for managing dysphagia in neurological conditions.

Parkinson's disease (PD) is another extensively studied condition in TMS research. Wang et al. investigated reactive motor inhibition deficits in PD by examining the interaction between the right dorsolateral prefrontal cortex (DLPFC) and the left primary motor cortex (M1) using paired-pulse TMS (ppTMS) in PD patients and healthy controls. The study revealed that PD patients exhibited longer stop-signal reaction times (SSRT) due to abnormal modulation of the right DLPFC–left M1 pathway, specifically enhanced inhibition following the stop-signal at short and long latencies. These results suggest that impaired reactive inhibition in PD stems from abnormal DLPFC-M1 interaction during reactive stopping, offering insights into motor dysfunction in PD and potentially informing neuromodulatory treatments.

In the application of TMS, MEP measurements are particularly important as they provide valuable insights into the status of the corticospinal system, changes in neural circuitry associated with neurological conditions, and the underlying neurophysiological mechanisms of rTMS protocols. Valente et al. emphasized the importance of the placement and dimensions of the surface electromyography (EMG), highlighting their impact on MEP properties. Recent applications of high-density surface EMG in measuring MEP offers the potential to improve the reliability of MEP recordings and detect subtle changes in the muscle activation patterns.

TMS has undoubtedly contributed to advancing our understanding of motor control and motor rehabilitation. Ongoing research is crucial for further deepening our knowledge and optimizing therapeutic approaches to advance the field.
